# Lactate Transporters Mediate Glia-Neuron Metabolic Crosstalk in Homeostasis and Disease

**DOI:** 10.3389/fncel.2020.589582

**Published:** 2020-09-29

**Authors:** Mithilesh Kumar Jha, Brett M. Morrison

**Affiliations:** Department of Neurology, Johns Hopkins University School of Medicine, Baltimore, MD, United States

**Keywords:** monocarboxylate transport, neurodegenerative disease, Schwann cell, oligodendrocyte, astrocyte, peripheral nerve, lactate

## Abstract

Research over the last couple of decades has provided novel insights into lactate neurobiology and the implications of lactate transport-driven neuroenergetics in health and diseases of peripheral nerve and the brain. The expression pattern of lactate transporters in glia and neurons has now been described, though notable controversies and discrepancies remain. Importantly, down- and up-regulation experiments are underway to better understand the function of these transporters in different systems. Lactate transporters in peripheral nerves are important for maintenance of axon and myelin integrity, motor end-plate integrity, the development of diabetic peripheral neuropathy (DPN), and the functional recovery following nerve injuries. Similarly, brain energy metabolism and functions ranging from development to synaptic plasticity to axonal integrity are also dependent on lactate transport primarily between glia and neurons. This review is focused on critically analysing the expression pattern and the functions of lactate transporters in peripheral nerves and the brain and highlighting their role in glia-neuron metabolic crosstalk in physiological and pathological conditions.

## Introduction

Though reported two centuries ago in exhausted animal muscle as a waste product, recent evidence and new lines of investigation have redefined the biology of lactate (L-lactate or lactic acid). Lactic acid, which is hydrophilic and a weak acid, donates hydrogen ions (H^+^) and the resultant product, a hydroxy monocarboxylic acid anion, referred to as lactate, is the conjugate base of lactic acid. Pyruvate under both anaerobic and aerobic (Warburg effect; Warburg et al., 1927) conditions is metabolized by lactate dehydrogenase (LDH) to lactic acid. Importantly, the nervous system operates mainly under aerobic conditions but cannot fully oxidize glucose and instead generates a local surplus of lactate, a phenomenon termed aerobic glycolysis (Barros et al., 2020). In aqueous solutions, lactic acid dissociates almost completely to lactate and H^+^. In nature, lactate exists in two isomers: L-lactate and D-lactate, with L-lactate being by far the most abundant and physiologically significant in all mammals, including humans. Recent studies have recognized lactate as a major carbon source fueling metabolic pathways and a key molecule regulating diverse biological processes (Faubert et al., 2017; Hui et al., 2017; Brooks, 2018; Magistretti and Allaman, 2018). Growing evidence now acknowledges lactate as an active metabolite capable of moving into or out of cells, acting as a signaling molecule, and regulating diverse physiological and pathophysiological cascades.

Studies over the past 20 years have defined the cellular metabolic heterogeneity of the nervous system, particularly in the brain. Metabolically, neurons are mostly oxidative, whereas glial cells, particularly astrocytes and oligodendrocytes, are predominantly glycolytic, and thus metabolize glucose to lactate to a greater extent than neurons (Funfschilling et al., 2012; Magistretti and Allaman, 2015, 2018; Weber and Barros, 2015; Supplie et al., 2017). Neurons can use lactate from astrocytes as an energy substrate particularly during functional activation, suggesting that brain energy metabolism begins as transient glycolysis in astrocytes and ends as oxidation in neurons. The lactate: pyruvate concentration ratio of 10:1 under physiological conditions indicates that lactate is the predominant glycolytic substrate for intercellular transfer in the brain (Alberti, 1977; Levy, 2006; Redant et al., 2019). Similarly, emerging evidence suggests a functional intercellular lactate shuttle in peripheral nerves (Takebe et al., 2008; Brown et al., 2012; Evans et al., 2013; Saab et al., 2013; Tu et al., 2017). Schwann cells contain glycogen, which is metabolized to lactate that can substitute for glucose in supporting axonal functions and survival (Brown et al., 2012). Besides having a metabolic role, a growing body of evidence demonstrates that lactate functions as an important signaling molecule in various tissues and cell types under physiological and pathological conditions and contributes to several homeostatic processes (Cha et al., 1991; Brooks, 2002; Hirschhaeuser et al., 2011; Jha et al., 2015, 2016; Rahman et al., 2016; Magistretti and Allaman, 2018).

Lactate transport across membranes requires monocarboxylate transporters (MCTs) of the SLC16 solute carrier family (Halestrap, 2013b). MCTs are a family of proton-linked plasma membrane transporters that allow the passage of monocarboxylates, including lactate, pyruvate, and ketone bodies (Halestrap and Price, 1999; Halestrap and Meredith, 2004; Bosshart et al., 2019). Though there are 14 members of this family, only the first four (MCT1-4) have been recognized experimentally to transport metabolically important monocarboxylates such as lactate, pyruvate and ketone bodies, each with distinct substrate and inhibitors affinities. However, the key substrates for most of the other MCTs are different from that of MCTs 1–4 or are still unknown [for review see (Jones and Morris, 2016)]. MCTs display distinct affinities for these monocarboxylates and are differentially expressed within cells and tissues. Although Camillo Golgi proposed that glial cells are metabolic supporters for neurons based on his microscopic observations over a century ago [(Golgi, 1873) as reviewed in Parpura and Verkhratsky (2012)], the metabolic interactions and nutrient sharing between glia and neurons are just now starting to be understood. Experiments completed in the last couple of decades have led to several important breakthroughs regarding the metabolic crosstalk between glia and neurons. This review focuses on summarizing the emerging roles of lactate transporters in glia-neuron metabolic interactions in the central and peripheral nervous systems (CNS and PNS, respectively) and exploring the therapeutic potentials of targeting the lactate transporter pathways for neurological disorders.

## Differentially Expressed Lactate Transporters Establish Glia-Neuron Metabolic Crosstalk

In human and other mammalian cells, transport of L-lactate across plasma membranes is mainly catalyzed by proton-linked MCTs of the SLC16 solute carrier family. Sodium-coupled MCTs (SMCTs) can also function as L-lactate transporters, though the function for these transporters in the nervous system remains unknown.

Each of these MCTs (1–4) exhibits a distinct regional and cellular distribution (Tables 1, 2). MCT3 expression is restricted to retinal pigmented epithelial cells (Philp et al., 1998) and choroid plexus epithelium (Philp et al., 2001), but the other three transporters are all expressed in the CNS and PNS (Debernardi et al., 2003; Pellerin et al., 2005; Pierre and Pellerin, 2005; Lee et al., 2012; Halestrap, 2013b; Nijland et al., 2014; Domenech-Estevez et al., 2015; Morrison et al., 2015; Perez-Escuredo et al., 2016; Jha and Morrison, 2018; Mornagui et al., 2019). In the CNS, MCT1 is expressed in oligodendrocytes (Rinholm et al., 2011; Lee et al., 2012; Morrison et al., 2013), astrocytes (Broer et al., 1997; Leino et al., 1999; Hanu et al., 2000; Tseng et al., 2003; Pellerin et al., 2005; Tekkok et al., 2005; Chiry et al., 2006; Nijland et al., 2014), microglia (Moreira et al., 2009; Ding et al., 2013; Nijland et al., 2014; Kong et al., 2019), endothelial cells (Gerhart et al., 1997; Pellerin et al., 1998b; Mac and Nalecz, 2003; Tseng et al., 2003; Chiry et al., 2006; Balmaceda-Aguilera et al., 2012), tanycytes (hypothalamus-specific glial cell type) (Tseng et al., 2003; Cortes-Campos et al., 2011), ependymocytes (Tseng et al., 2003), and some specific neurons (Tseng et al., 2003; Chiry et al., 2006; Balmaceda-Aguilera et al., 2012; Morrison et al., 2013; Perez-Escuredo et al., 2016). Similarly, in the PNS, MCT1 is expressed in perineurial cells (Takebe et al., 2008; Morrison et al., 2015) and endoneurial cells, including Schwann cells (Domenech-Estevez et al., 2015; Morrison et al., 2015; Jha et al., 2020b) and DRG neurons (Domenech-Estevez et al., 2015; Morrison et al., 2015). Though clearly expressed in the PNS (Domenech-Estevez et al., 2015; Morrison et al., 2015), the precise cellular localization of MCT2 in the PNS is still unclear. In the CNS, it is expressed predominately in neurons (Tekkok et al., 2005; Chiry et al., 2006; Balmaceda-Aguilera et al., 2012; Nijland et al., 2014; Alvarez-Flores et al., 2019), though other studies have shown expression in endothelial cells (Mac and Nalecz, 2003; Chiry et al., 2006; Balmaceda-Aguilera et al., 2012), astrocytes (Gerhart et al., 1998; Hanu et al., 2000; Nijland et al., 2014), microglia (Moreira et al., 2009; Nijland et al., 2014; Kong et al., 2019), and tanycytes (Cortes-Campos et al., 2011). MCT4 expression in the CNS is very low and is expressed mostly in astrocytes (Marcillac et al., 2011; Lee et al., 2012; Nijland et al., 2014; Rosafio and Pellerin, 2014), though lower levels have been found in microglia (Nijland et al., 2014; Kong et al., 2019), tanycytes (Cortes-Campos et al., 2011), and endothelial cells (Balmaceda-Aguilera et al., 2012; Nijland et al., 2014). In the PNS, MCT4 is expressed in Schwann cells (Domenech-Estevez et al., 2015).

**TABLE 1:** Regional and cellular distribution of MCTs in the peripheral nervous system.

MCTs	Cells	Species	Methods	References
MCT1	Perineurial cells	Mouse	qPCR, WB, ISH, BAC, or IHC	Takebe et al., 2008; Morrison et al., 2015
	Schwann cells	Mouse and rat	qPCR, WB, BAC, or IHC	Domenech-Estevez et al., 2015; Morrison et al., 2015; Jha et al., 2020b
	DRG neurons	Mouse and rat	qPCR, WB, BAC, or IHC	Domenech-Estevez et al., 2015; Morrison et al., 2015
MCT2	Expressed in the PNS, but cellular distribution is still unclear	Mouse and rat	qPCR, WB, or IHC	Domenech-Estevez et al., 2015; Morrison et al., 2015
MCT4	Schwann cells	Mouse and rat	qPCR, WB, or IHC	Domenech-Estevez et al., 2015

*qPCR, real-time RT PCR; NB, Northern blot; WB, Western blot; ISH, In situ hybridization; BAC; BAC transgenic reporter; IHC, Immunohistochemistry.*

**TABLE 2:** Regional and cellular distribution of MCTs in the central nervous system.

MCTs	Cells	Species	Methods	References
MCT1	Oligodendrocytes	Mouse, rat, and human	qPCR, WB, BAC, or IHC	Rinholm et al., 2011; Lee et al., 2012; Morrison et al., 2013
	Astrocytes	Mouse, rat, and human	WB, NB, or IHC	Broer et al., 1997; Leino et al., 1999; Hanu et al., 2000; Tseng et al., 2003; Tekkok et al., 2005; Chiry et al., 2006; Nijland et al., 2014
	Microglia	Mouse, rat, and human	qPCR, WB, or IHC	Moreira et al., 2009; Ding et al., 2013; Nijland et al., 2014; Kong et al., 2019
	Endothelial cells	Rat, shark, and human	NB, IHC, ISH, or RT-PCR	Gerhart et al., 1997; Pellerin et al., 1998b; Mac and Nalecz, 2003; Tseng et al., 2003; Chiry et al., 2006; Balmaceda-Aguilera et al., 2012
	Ependymal cells (tanycytes and ependymocytes)	Rat	qPCR or IHC	Tseng et al., 2003; Cortes-Campos et al., 2011
	Neurons (some specific neurons)	Rat, shark, and human	IHC	Tseng et al., 2003; Chiry et al., 2006; Balmaceda-Aguilera et al., 2012
MCT2	Endothelial cells	Rat, shark, and human	qPCR or IHC	Mac and Nalecz, 2003; Chiry et al., 2006; Balmaceda-Aguilera et al., 2012
	Neurons	Shark and human	IHC	Tekkok et al., 2005; Chiry et al., 2006; Balmaceda-Aguilera et al., 2012; Nijland et al., 2014; Alvarez-Flores et al., 2019
	Astrocytes	Rat and human	WB or IHC	Gerhart et al., 1998; Hanu et al., 2000; Nijland et al., 2014
	Microglia	Mouse, rat, and human	IHC	Moreira et al., 2009; Nijland et al., 2014; Kong et al., 2019
	Tanycytes	Rat	qPCR or IHC	Cortes-Campos et al., 2011
MCT3	Retinal pigmented epithelial cells	Rat	IHC, WB, and NB	Philp et al., 1998
	Choroid plexus epithelium	Rat	IHC, WB, and NB	Philp et al., 2001
MCT4	Astrocytes	Mouse, rat, and human	qPCR, WB, BAC, and IHC	Marcillac et al., 2011; Lee et al., 2012; Nijland et al., 2014; Rosafio and Pellerin, 2014
	Microglia	Mouse and human	IHC	Nijland et al., 2014; Kong et al., 2019
	Tanycytes	Rat	qPCR and IHC	Cortes-Campos et al., 2011
	Endothelial cells	Shark and human	IHC	Balmaceda-Aguilera et al., 2012; Nijland et al., 2014

*qPCR, real-time RT PCR; NB, Northern blot; WB, Western blot; ISH, In situ hybridization; BAC, BAC transgenic reporter; IHC, Immunohistochemistry.*

Besides the proton-linked co-transporters of monocarboxylic substrates, there is a second class of MCTs known as sodium-coupled MCTs (SMCTs). This class of MCTs contains two members, namely SMCT1 (SLC5A8) and SMCT2 (SLC5A12), that mediate cellular uptake of monocarboxylates in a sodium (Na^+^)-coupled manner (Ganapathy et al., 2008; Srivastava et al., 2019). SMCTs, which depend on a sodium gradient for their functional activity, act as a symporter and play an important role in handling multiple endogenous monocarboxylates in various tissues throughout the body (Ganapathy et al., 2008; Halestrap, 2013a; Lu et al., 2013; Vijay and Morris, 2014; Iwanaga and Kishimoto, 2015). SMCT1 is a high-affinity, whereas SMCT2 is a low-affinity, lactate transport system. Both are expressed in the brain and retina. SMCT1 is restricted to neurons and retinal pigment epithelium and contributes to cellular uptake of lactate in neurons (Martin et al., 2006). The expression pattern of SMCT1 is similar to that of neuron-specific MCT2 (Ganapathy et al., 2008). The expression of low-affinity SMCT2 is restricted to astrocytes and Müller cells, the glial cells of the retina (Martin et al., 2007). Physiologically, SMCT1 can transport lactate and ketone bodies into neurons and also functions as a tumor suppressor in the brain, but the importance of this transporter in normal physiologic conditions is unknown (Ganapathy et al., 2008). The function of SMCT2 in the nervous system has been even less explored.

## Lactate Transporter-Dependent Glial Metabolism Supports Peripheral Nerve Integrity and Function

Integrity and function of the PNS depends upon uninterrupted energy supply. Although the transfer of metabolic substrates form Schwann cells to axons was reported about two decades ago (Vega et al., 2003), the specific mechanism behind the metabolic transfer in the PNS is still unclear. Emerging evidence suggests that lactate is a preferred and an effective energy source for the PNS, and the lactate shuttle, similar to that in the CNS (Pellerin et al., 1998a; Magistretti, 2006; Belanger et al., 2011; Magistretti, 2011), also functions in the PNS through the differential expression of MCTs in PNS cells (Domenech-Estevez et al., 2015; Morrison et al., 2015; Jha and Morrison, 2018; Jha et al., 2020b). Earlier studies suggest that Schwann cells contain glycogen, which can be metabolized to lactate and substitute for glucose in maintaining axonal function and survival via a lactate transporter-mediated mechanism (Brown et al., 2012). Some recent studies from different laboratories, including ours, document that MCT1 is the primary and most abundantly expressed lactate transporter in peripheral nerves, and is crucial for neuron-glia metabolic coupling in the PNS (Takebe et al., 2008; Domenech-Estevez et al., 2015; Morrison et al., 2015; Jha and Morrison, 2018; Jha et al., 2020b).

**FIGURE 1:**
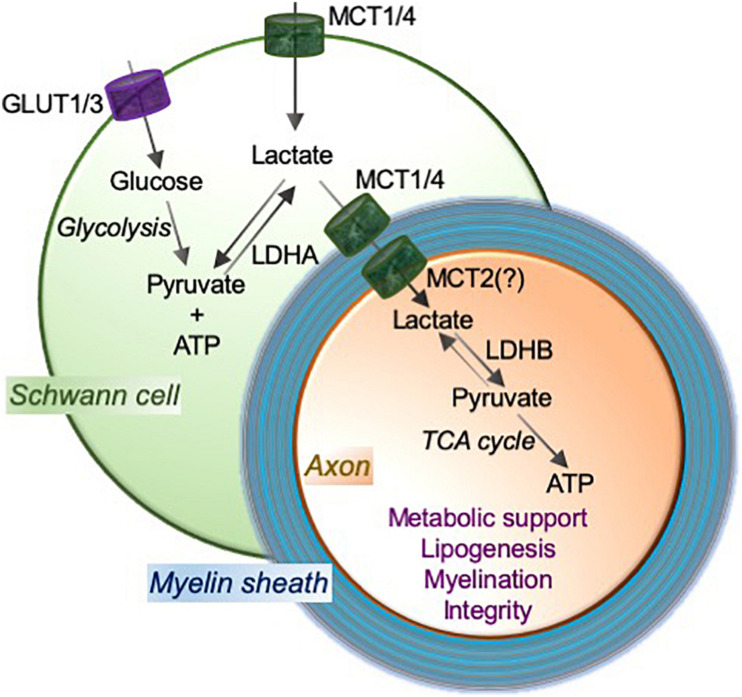
MCTs mediate the lactate shuttle between Schwann cells and neurons in the peripheral nervous system. Schwann cell-imported glucose through the glucose transporters GLUT1 and GLUT3 is metabolized to pyruvate and ATP by glycolysis, and pyruvate to lactate using a glycolytic enzyme lactate dehydrogenase (LDH; especially LDHA). Schwann cells employ monocarboxylate transporters MCT1 and MCT4 to import and/or export lactate. MCT2, or potentially other transporter, functions to import lactate into axons. LDH (especially LDHB) oxidizes lactate to pyruvate that can provide substrates to the tricarboxylic acid (TCA) cycle, which results in adenosine triphosphate (ATP) production through oxidative phosphorylation. This Schwann cell to neuron lactate shuttle and subsequent downstream metabolic pathways provide metabolic support and contribute critically to lipogenesis and myelination, which are essential for axonal function and integrity.

MCT1 has been found to be crucial for Schwann cell biology and it contributes to both myelin maintenance (Jha et al., 2020b) and neuromuscular innervation (Boucanova et al., 2020). These recent studies support the notion that MCT1 modulates the metabolic support from Schwann cells to axons and is essential for normal peripheral nerve physiology. We have recently evaluated the impact of Schwann cell-specific MCT1 ablation on cell biology, peripheral nerve metabolism, and the integrity and function of peripheral nerves during development and aging (Figure 1). Ablation of MCT1 only in Schwann cells is found to significantly reduce its expression in the whole sciatic nerve, indicating that Schwann cells are one of the major MCT1 producing cells in the peripheral nerve (Jha et al., 2020b). MCT1 ablation in Schwann cells impairs glycolytic and mitochondrial functions, depletes the nerve of critical lipids, especially sphingomyelins, diacylglycerides, and triacylglycerides, and results in hypomyelination (Jha et al., 2020b). Hypomyelination was detected specifically in sensory nerves both by electrophysiology (i.e., slowed nerve conduction studies) as well as histologically. Functionally, mice with Schwann cell-specific MCT1 deficiency show deficits in sensory, but not motor, peripheral nerves during aging (Jha et al., 2020b). We did not observe any significant compensatory alteration in the expression of other MCTs (i.e., MCT2 and MCT4) following Schwann cell-specific MCT1 ablation, likely reflecting the subtle role of Schwann cell MCT1 in maintaining myelination that does greatly impact development or animal survival. Interestingly, a recent study suggests significant reduction of MCT1 in peripheral myelin of periaxin-deficient mice, which is a model of inherited Charcot-Marie-Tooth 4F peripheral neuropathy (Siems et al., 2020). These mice lacking periaxin develop clear evidence of impaired axonal integrity, such as reduced axonal diameters, a progressively reduced total number of axons, and a considerable number of myelin whorls lacking a visible axon. A more recent study has also characterized the impact of Schwann cell-specific MCT1 or MCT4 ablation on nerve biology during development (Boucanova et al., 2020). Consistent with our findings (Jha et al., 2020b), this study reports normal development of the PNS in Schwann cell-specific MCT1 deficient mice. This study also reports no notable impact of Schwann cell-specific MCT4 deletion on PNS development. Potentially due to differences in genetic background or quantification of myelination from different nerves (e.g., sciatic vs. sural nerve), this study found no impact of Schwann cell-specific MCT1 ablation on sensory nerve conduction velocity or myelination (Boucanova et al., 2020), which clearly differs from our study, as described above (Jha et al., 2020b). This study also reported that Schwann cell-specific MCT1 is necessary for long-term maintenance of motor end-plate integrity, which was not investigated in our Schwann cell-specific MCT1 null mice, while MCT4 appears largely dispensable for the support of motor neurons (Boucanova et al., 2020). Taken together, both studies demonstrate important, though different, roles of the lactate transporter MCT1 in Schwann cell function and metabolic interactions with axons during development and aging.

Lactate transport through MCT1 in the PNS also has an essential role in axonal regeneration following injury, potentially through metabolic support from Schwann cells to axons (Morrison et al., 2015). Consistent with an earlier study using sciatic nerve explants to demonstrate the dependence of axons on lactate for metabolic energy (Brown et al., 2012), we demonstrated that axons in an injured condition, specifically following crush of the sciatic nerve, depend on MCT1 for transport of lactate as an energy substrate (Morrison et al., 2015). This study demonstrates that MCT1 is critical for peripheral nerve regeneration and its deficiency delays the recovery following sciatic nerve injury in mice. Given that these experiments were completed in heterozygous MCT1 null mice, which have a 50% reduction of MCT1 in all cell types, the role of MCT1 during regeneration in individual cell types remains to be determined.

Diabetic peripheral neuropathy (DPN) is the most common complication of diabetic patients and it involves metabolic dysfunction and energy failure in the PNS. Our most recent studies suggest a critical role of lactate transporter MCT1 in the pathogenesis of DPN (Jha et al., 2020a). MCT1 expression in the PNS, both peripheral nerve and the dorsal root ganglion, of mice is decreased after diabetic induction. Employing heterozygous MCT1 null mice, as described above, we have found that mice with reduced expression of MCT1 develop more severe DPN compared to wild-type mice following streptozotocin injection (Jha et al., 2020a). Streptozotocin is an alkylating agent toxic to insulin-producing pancreatic beta cells. Streptozotocin injection induces hypoinsulinemia and chronic hyperglycemia, mimicking type 1 diabetes phenotypes in mice. MCT1 heterozygous null mice after diabetes induction develop greater axonal demyelination, decreased peripheral nerve function as measured by electrophysiology, and increased numbness to innocuous low-threshold mechanical stimulation, suggesting an important role of MCT1 in the development of DPN. Though the mechanism is still to be explored, the findings of this study, along with others (Morrison et al., 2015; Feldman et al., 2017; Jha and Morrison, 2018; Jha et al., 2020b; Siems et al., 2020) support an important role for predominately glycolytic Schwann cells to supply metabolic energy to axons during development and certain disease models.

## Brain Energy Metabolism and Function Critically Depend on Glia-Neuron Lactate Dynamics

**FIGURE 2:**
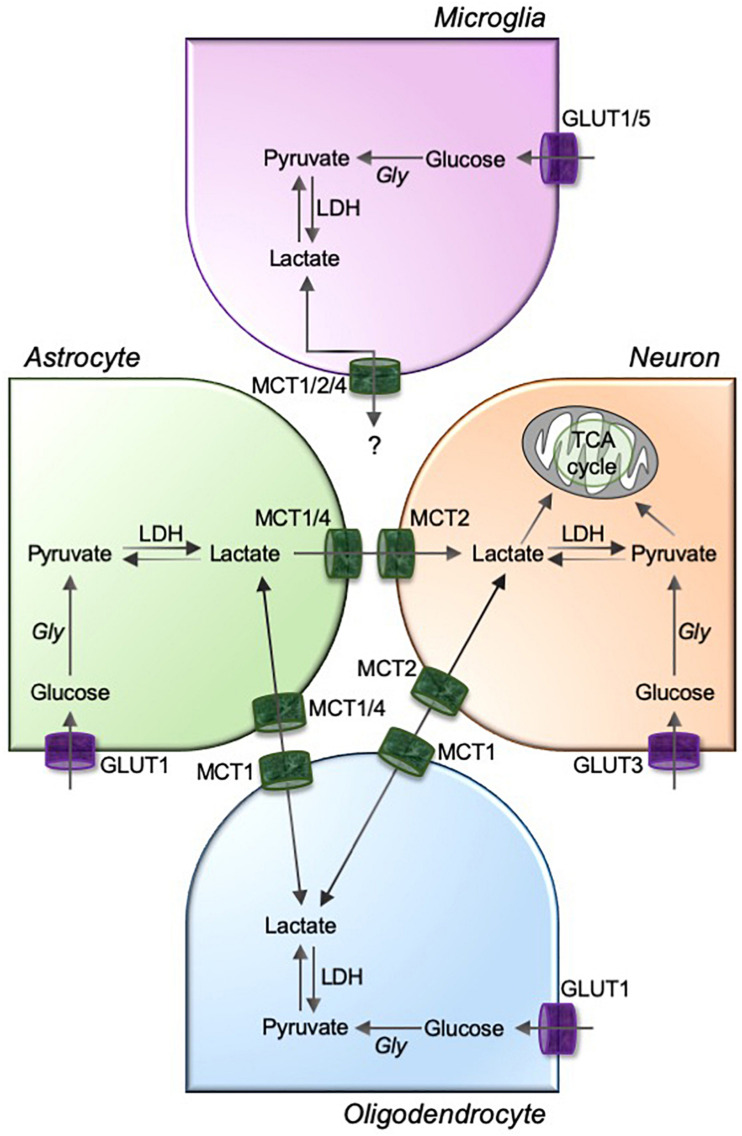
MCTs mediate the lactate exchanges between glia and neurons in the brain. Astrocyte, oligodendrocytes, and microglia are highly glycolytic cells that take up circulating glucose through glucose transporters (GLUTs; GLUT1 for astrocytes and oligodendrocytes, and GLUT1 and GLUT5 for microglia). GLUT3 facilitates neuronal uptake of glucose. The glucose is metabolized to pyruvate using glycolysis, and pyruvate to lactate using a glycolytic enzyme lactate dehydrogenase (LDH). Although unknown for microglia, astrocyte and oligodendrocyte-derived intracellular lactate is transported to neurons through a pathway involving monocarboxylic acid transporters, MCT1/4 and MCT2, as depicted in figure. Gly, glycolysis; TCA, tricarboxylic acid.

Glia-neuron metabolic coupling in the CNS is primarily mediated by lactate shuttling through MCTs, and the expression of MCTs varies during development and under the influence of neurotransmitters and nutritional modifications, indicating its pivotal role in brain energy metabolism and functions (Pellerin et al., 1998b). Most of the neuronal and glial cells in the CNS differentially express MCTs (Table 2). Glial cells produce lactate from glycogen stores or glucose via glycolytic metabolism (Cortes-Campos et al., 2011). MCT1 is a bi-directional transporter that is highly expressed in astrocytes (Broer et al., 1997; Leino et al., 1999; Hanu et al., 2000; Tseng et al., 2003; Tekkok et al., 2005) and oligodendrocytes (Rinholm et al., 2011; Lee et al., 2012). Therefore, MCT1 may be important in these cell types both for importing lactate that can ultimately be metabolized in the TCA cycle or exporting lactate to clear this end product of glycolysis. It is now well accepted that exported glial lactate can provide metabolic energy to surrounding neuron and axons, primarily by being metabolized in the TCA cycle/oxidative phosphorylation (Jha and Morrison, 2018), and contribute to the maintenance of axonal myelination and neuronal integrity (Debernardi et al., 2003; Belanger et al., 2011; Harris and Attwell, 2012; Saab et al., 2016; Descalzi et al., 2019). In contrast to MCT1, MCT2 is highly expressed in neurons. This cell-specific expression of MCTs in the CNS facilitates the proposed glia-neuron lactate shuttle (Figure 2), which depends on lactate being produced and released from astrocytes and oligodendrocytes (via MCT1 or possibly MCT4) and taken up by neurons (via MCT2) during neuronal activities (Pierre et al., 2000; Funfschilling et al., 2012; Lee et al., 2012; Morrison et al., 2013). The kinetics of neuronal MCT2 and LDH1 and astrocytic MCT1/4 and LDH5 supports the astrocytic production and neuronal consumption of lactate (Bittar et al., 1996; Laughton et al., 2000; Debernardi et al., 2003; Pierre and Pellerin, 2005; Machler et al., 2016). Although the role of microglia in metabolite sharing in the brain has not been as comprehensively studied, recent publications propose an astrocyte-microglia lactate shuttle during chronic neuroinflammatory infectious diseases (Mason et al., 2015; Mason, 2017). Utilizing proton magnetic resonance (^1^H NMR)-based metabolomics analysis and several chemometric methods in lumbar cerebrospinal fluid samples, this study suggests that astrocytes respond to signaling from *Mycobacterium tuberculosis*-infected microglia by increasing glucose metabolism that ultimately leads to increased extracellular lactate in the cerebrospinal fluid. This astrocyte-derived lactate may subsequently be used by microglia as an energy source for the production of reactive oxygen to destroy the invading *Mycobacterium tuberculosis* (Mason et al., 2015; Mason, 2017). Similarly, microglial activation with lipopolysaccharide and interferon-γ has been proposed to activate a microglia-astrocyte-neuron lactate shuttle, particularly in response to excitotoxic stimuli (Gimeno-Bayon et al., 2014).

Astrocyte-neuron metabolic coupling established mostly through MCTs is the underlying molecular mechanism for the astrocyte-neuron lactate shuttle and brain energetic support (Pellerin et al., 1998a; Magistretti et al., 1999; Whitlock et al., 2006; Herrero-Mendez et al., 2009; Belanger et al., 2011). During periods of high energy demand, glycogen stored in astrocytes is metabolized to lactate and shuttled to neurons though MCTs (Pellerin and Magistretti, 1994; Suzuki et al., 2011). Besides providing this neuronal energy support, MCTs and lactate play an active role in neural and synaptic plasticity and function, learning, and memory (Dringen et al., 1993; Brown et al., 2004; Gibbs et al., 2006; Jahanshahi et al., 2008; Halassa and Haydon, 2010; Henneberger et al., 2010; Bezzi and Volterra, 2011; Suzuki et al., 2011; Santello et al., 2019; Ding et al., 2020; Murphy-Royal et al., 2020; Netzahualcoyotzi and Pellerin, 2020). Cognitive dysfunction and learning and memory impairment are found to be associated with altered expression of MCTs (Leroy et al., 2011; Lauritzen et al., 2012; Perez-Escuredo et al., 2016). Recent studies have also demonstrated that learning and memory deficits are observed in rats following inhibition of hippocampal MCT1 and MCT4 (Suzuki et al., 2011; Sun et al., 2018; Descalzi et al., 2019). Similarly, expression knockdown of MCT2, which is selectively expressed by neurons, impairs memory, suggesting the critical role of astrocytic lactate to provide energy for neuronal responses, including learning-induced mRNA translation in both excitatory and inhibitory neurons, required for long-term memory (Descalzi et al., 2019).

The glia-neuron lactate shuttle in the CNS has been found to be crucial for axonal myelination and integrity. Like Schwann cells in the PNS, oligodendrocytes are the cells that make myelin to ensheath neuronal axons in the CNS. This process, which requires large energy stores to maintain cell function and produce the lipids and myelin proteins, depends predominantly on lactate metabolism as a fuel (Sanchez-Abarca et al., 2001). In fact, earlier studies suggest that lactate supports myelination *in vitro* in the setting of glucose deprivation (Rinholm et al., 2011). Several studies have previously reported higher expression of MCT1 in the CNS myelin than in axons and conversely higher expression of MCT2 in axons than myelin (Rinholm et al., 2011; Lee et al., 2012; Jha and Morrison, 2018). Recent studies also report that oligodendrocyte progenitor cells metabolize glycogen to lactate and that lactate is transported through MCTs to promote cell cycling and differentiation in oligodendrocyte progenitor cell-rich culture (Ichihara et al., 2017). Given these findings, it is not surprising that the expression of MCTs is altered in multiple sclerosis (MS), which is the most common demyelinating disease of the CNS. MCT1 expression is increased in infiltrating leukocytes and reactive astrocytes in active MS lesions, and MCT2 expression is decreased in inactive MS lesions (Nijland et al., 2014). The loss of MCT2 in MS brains certainly may be from neuronal loss, and further experiments are necessary to determine if the expression changes precede neuronal death. If MCT and lactate changes occur early in MS, the deficiency of lactate supply to hypoxic demyelinated axons may contribute to neuronal degeneration in MS. These findings suggest that targeting lactate transport through MCTs can be a promising strategy for exploring therapeutics to promote remyelination in diverse demyelinating neurologic disease, including multiple sclerosis and inherited leukodystrophies.

Emerging evidence suggests a strong correlation between disruption of MCTs and neurodegeneration, particularly in amyotrophic lateral sclerosis (ALS) and Alzheimer’s disease (AD). Our earlier studies demonstrate that MCT1 expression is reduced in the motor cortex of ALS patients and spinal cord of the SOD1^G93A^ ALS rodent models (Lee et al., 2012). In this publication, we also found that MCT1 is highly enriched within oligodendrocytes and that transgenic or viral-mediated reduction of MCT1 either globally or selectively in oligodendrocytes causes axonal damage and neuronal loss in animal and cell culture models. These results suggest that oligodendroglia-specific MCT1 plays a role in supporting axons and that disruption of this support may contribute to motor neuron degeneration in ALS. Similarly, MCT1 expression also declines in both aging and Alzheimer’s disease (AD) (Ding et al., 2013). Furthermore, both MCT2 expression and lactate content are reduced in the cerebral cortex and hippocampus of a rat model of AD, suggesting impairment of lactate transport and energy metabolism in the AD brain (Lu et al., 2015). It is still not completely known, however, whether alterations in MCT expression is a primary event that contributes to neurodegeneration or a secondary event that results from glial changes or neuronal loss. Interestingly, MCTs appear to be altered early in patients at risk for developing AD since young asymptomatic adult carriers of the apolipoproteins E ε4 allele (APOE4), who are at high risk for developing AD, have increased expression of MCT2 and decreased expression of MCT4 in posterior cingulate cortex, as measured by Western blot analysis (Perkins et al., 2016). Additionally, a recent study found that astrocytic MCT4 is increased in the hippocampus of a commonly used mouse model of AD and overexpression in cultured astrocytes reduces neurite outgrowth and increases apoptosis of primary neurons in a co-culture model (Hong et al., 2020). In the same AD mouse model, viral delivery of MCT4 siRNA improves their cognitive phenotype. Here, the improvement following downregulation of MCT4 appears counterintuitive to the lactate shuttling hypothesis and may involve stimulating second messenger pathways within cells. Though the exact mechanism has not been elucidated in these paradigms, these results suggest that MCTs play a role in the development of AD and that targeting MCTs may provide an avenue for the development of novel therapies. The changes in ALS and AD are not found universally in all neurodegenerative diseases, however, since neither the expression of MCT1 or MCT2, nor the content of lactate, is altered in the substantia nigra and striatum in an experimental mouse model of Parkinson’s disease (Puchades et al., 2013). Though the contribution of MCTs to human neurological diseases still requires further study, the published studies are very provocative and suggest that MCTs are critical for the maintenance of neuronal integrity and function in the setting of neurologic disease.

## Controversies Related to Lactate and Its Transporters in the Nervous System

Though improving in recent years, the cellular/tissue-specific distribution of lactate transporters and their functions in both physiological and pathological conditions remains controversial and highly debated. The cellular expression of MCTs discussed above (Tables 1, 2) should be cautiously analyzed since most of them are not completely validated *in vivo* by proper knockout studies. Future studies are critically needed to fully clarify the functional expression of MCTs in the nervous system. The comprehensive understanding of any biomolecule needs investigations employing specific antibodies and pharmacological inhibitors as well as genetic tools. Currently, there are only few available antibodies against these MCTs, and their specificities, in many cases, are highly questionable. Similarly, there is no specific pharmacological antagonist or agonist for any of these MCTs. For this reason, the recent development of conditional knockout mice for MCT1 and MCT4 is critical for investigating the cell-specific role for these transporters *in vivo* (Boucanova et al., 2020; Jha et al., 2020b). Further development of these immunologic, pharmacological, and genetic tools will be critical in the future for clarifying the controversies surrounding the lactate transporters and lactate functions in the nervous system.

Though it was proposed about 30 years ago, the glia to neuron, especially astrocyte-neuron, lactate shuttle hypothesis remains controversial and is not fully accepted (Magistretti et al., 1993; Pellerin and Magistretti, 1994). There is still debate on whether, and in what conditions, lactate is used as the preferential metabolic substrate by neurons. Neurons are reported to utilize their own glucose when cultured alone (Bak et al., 2009). In fact, a study using simultaneous measurements of electrophysiological and metabolic parameters during synaptic stimulation in hippocampal slices from mature mice suggest that neurons use both glycolysis and oxidative phosphorylation to meet their energy demands, indicating that glucose, but not lactate released from astrocytes, is an effective energy substrate for neurons (Ivanov et al., 2014). Furthermore, one study suggests that neuronal stimulation, at least in the hippocampus, triggers neuronal glycolysis and the release of lactate from neurons (Diaz-Garcia et al., 2017). In contrast, many others provide evidence that lactate is released from astrocytes and delivered to neurons, both in response to cortical activation by arousal triggers (Zuend et al., 2020) or stimulation with cannabinoids (Jimenez-Blasco et al., 2020). As with most complicated systems in neuroscience, it is likely that all of these processes can occur depending on the exact stimulus, environment, and cells involved. The inability to visualize lactate *in vivo* has complicated these studies, but perhaps further studies using lactate sensors, for example, laconic (San Martin et al., 2013), will help clarify these issues in the future.

## Discussion

Glial cells are now well acknowledged to be dynamic cells that sense metabolic needs of neurons and regulate their function by the transfer of energy metabolites. Lactate functions as a preferred and an effective energy source in the nervous system especially during high energy demands. Most glial cells, but especially astrocytes and oligodendrocytes in the CNS, and Schwann cells in the PNS, metabolize glucose to lactate and share it with neurons through MCTs. Differentially expressed MCTs are crucial for the establishment and functioning of the glia-neuron metabolic interactions in health and disease. Further investigations are necessary to confirm the contribution of these processes to human disease and to evaluate potential therapeutic strategies targeting lactate transporters for neurological disorders. Though preferential expression of MCTs occur in specific cell types, many of the MCTs, particularly MCT1, are expressed in numerous cell types in the nervous system. Hence, careful cell-specific studies will be necessary to better understand the exact mechanism of their transporter action. Cell-specific genetic ablation and high affinity pharmacological inhibitors/agonists are starting to be developed and more are necessary for the selective and controllable modulation of MCTs. These tools are highly awaited and will be critical to better understand the biology of lactate transport and glia-neuron metabolic interactions in health and diseases of nervous system.

## Author Contributions

MJ completed literature review and wrote the manuscript. BM secured funding and wrote the manuscript. All authors contributed to the article and approved the submitted version.

## Conflict of Interest

The authors declare that the research was conducted in the absence of any commercial or financial relationships that could be construed as a potential conflict of interest.
